# Efectos de los coronavirus del síndrome respiratorio agudo grave (SARS-CoV) y del síndrome respiratorio del Medio Oriente (MERS-CoV) en el sistema nervioso. ¿Qué esperar del SARS-CoV-2?

**DOI:** 10.7705/biomedica.5682

**Published:** 2020-11-15

**Authors:** Jeison Monroy-Gómez, Orlando Torres-Fernández

**Affiliations:** 1 Grupo de Neurociencias Aplicadas para la Salud y el Deporte, Grupo de Capacidades Humanas en Salud e Inclusión, Institución Universitaria Escuela Colombiana de Rehabilitación, Bogotá DC, Colombia Institución Universitaria Escuela Colombiana de Rehabilitación BogotáDC Colombia; 2 Grupo de Morfología Celular, Dirección de Investigación en Salud Pública, Instituto Nacional de Salud, Bogotá, D.C., Colombia Instituto Nacional de Salud BogotáD.C Colombia

**Keywords:** infecciones por coronavirus, síndrome respiratorio agudo grave, sistema nervioso, Coronavirus infections, severe acute respiratory syndrome, nervous system

## Abstract

Los coronavirus son una familia de virus que se caracterizan por producir afectaciones respiratorias y gastrointestinales en animales y en seres humanos. El actual SARS-CoV-2, agente infeccioso de la COVID-19, pertenece a un subgrupo denominado betacoronavirus del que hacen parte el SARS-CoV y MERS-CoV, virus responsables de epidemias en el 2002 y el 2012, respectivamente.

Estos virus también pueden infectar el sistema nervioso debido a su afinidad con la enzima convertidora de angiotensina humana 2 (ACE2), la cual se expresa en neuronas y células gliales. Se ha demostrado que las infecciones con SARS-CoV y MERS-CoV, y ahora también con el SARS-CoV-2, ocasionan condiciones neurológicas como la enfermedad cerebrovascular aguda, la conciencia alterada y las lesiones musculares, así como mareos, hipogeusia, hiposmia, hipoxia, neuralgia y encefalopatía hipóxica. Por ello debe prestarse mucha atención a las manifestaciones neurológicas de los pacientes de COVID-19.

## Abstract (en)

Coronaviruses cause respiratory and gastrointestinal disorders in animals and humans. The current SARS-CoV-2, the COVID-19 infectious agent, belongs to a subgroup called betacoronavirus including the SARS-CoV and MERS-CoV responsible for epidemics in 2002 and 2012, respectively.

These viruses can also infect the nervous system due to their affinity for the human angiotensin-converting enzyme 2 (ACE2) expressed in neurons and glial cells. Infections with SARS-CoV, MERS-CoV, and now SARS-CoV-2 also produce neurological signs such as acute cerebrovascular disease, impaired consciousness, and muscle injury, as well as dizziness, hypogeusia, hyposmia, hypoxia, neuralgia, and hypoxic encephalopathy. For this reason, close attention should be paid to the neurological manifestations of COVID-19 patients.

La familia de los coronavirus (Coronaviridae), del orden Nidovirales, se caracteriza por poseer ARN de sentido positivo, no segmentado y de tamaño grande. Causan infecciones respiratorias y gastrointestinales y, eventualmente, del sistema nervioso en animales y en humanos ^1^^,^^2^, aunque en estos los coronavirus (CoV) están asociados principalmente con enfermedades de las vías respiratorias ^3^. Los coronavirus se clasifican en cuatro grupos principales: alfacoronavirus, betacoronavirus, gammacoronavirus y deltacoronavirus. Los alfacoronavirus (HCoV-NL63 y HCoV-229E) y los betacoronavirus (HCoV-HKU1, SARS-CoV, MERS-CoV y HCoV-OC43) generalmente causan enfermedades respiratorias en humanos y gastroenteritis en animales ^1^^,^^4^. El actual SARS-CoV-2 hace parte de los betacoronavirus y su secuencia genética tiene más del 80 % de identidad con el SARS-CoV y del 50 % con el MERS-CoV ^5^^,^^6^.

El SARS-CoV y el MERS-CoV son dos de los virus más patógenos entre los betacoronavirus y causan un síndrome respiratorio grave en humanos; los otros cuatro coronavirus reportados en humanos (HCoV-NL63, HCoV-229E, HCoV-OC43 y HKU1) solo producen leves enfermedades respiratorias en las vías superiores en personas inmunocompetentes, aunque algunos pueden causar infecciones graves en infantes y personas de edad avanzada como la bronquiolitis y la neumonía y ser potencialmente mortales ^1^^,^^4^^,^^7^. Estos virus también pueden exacerbar el asma y provocar varios tipos de síndromes de dificultad respiratoria ^8^. Se ha reportado, asimismo, que algunos coronavirus pueden infectar el sistema nervioso central e inducir signos neurológicos _(9)_, de ahí el objetivo de esta actualización en el sentido de hacer una breve revisión de los efectos de las infecciones con SARS-CoV y MERS-CoV en el sistema nervioso y discutir lo que es posible esperar del SARS-CoV-2 en este campo.

## El síndrome respiratorio agudo grave y el SARS-CoV

El SARS-CoV surgió por primera vez entre el 2002 y el 2003 en Guangdong (China) y se extendió rápidamente a 27 países de Asia, Europa y América, con una tasa de mortalidad cercana al 10 %. Se reportaron 8.098 personas infectadas y 774 de ellas murieron ^2^. La fuente animal del SARS-CoV fueron las civetas, un grupo de pequeños mamíferos carnívoros, que se ofrecían en los mercados de vida silvestre en China y a las que los murciélagos habían transmitido el virus ^7^. Los síntomas del SARS inician con fiebre alta, dolor de cabeza, malestar y dolor en el cuerpo y, en algunos pacientes, diarrea; posteriormente puede presentarse tos seca. La mayoría de los pacientes contrae neumonía que puede evolucionar a un síndrome de dificultad respiratoria aguda potencialmente mortal ^4^^,^^7^.

En algunos pacientes se han reportado signos neurológicos como neuropatía olfativa (anosmia), neuropatía periférica y miopatía concomitante durante la etapa de convalecencia del SARS-CoV ^10^^,^^11^. En estudios patológicos *post mortem* en cerebros humanos se han hallado partículas virales en el líquido cefalorraquídeo y en el citoplasma de neuronas de la neocorteza y el hipotálamo, así como degeneración y necrosis neuronal, edema, hiperplasia de células gliales e infiltrados celulares ^12^^-^^14^. Además, se ha demostrado experimentalmente en ratones que el virus puede ingresar al sistema nervioso a través del bulbo olfatorio y la infección se disemina rápidamente a otras áreas encefálicas y circunvecinas generando inflamación perivascular grave y meningitis. En los experimentos la infección se logró gracias a que se utilizaron ratones transgénicos que expresaban la enzima convertidora de angiotensina humana 2 (ACE2). Este receptor, localizado principalmente en neuronas y células gliales, presenta gran afinidad con las partículas virales del SARS-CoV ^15^^,^^16^.

## El síndrome respiratorio del Medio Oriente y el MERS-CoV

La epidemia de MERS-CoV apareció en Arabia Saudita en el 2012 con una infección de signos clínicos similares a los del SARS-CoV, pero con una tasa de mortalidad mucho más alta, aproximadamente del 35 %. Hasta septiembre del 2016 se confirmaron 1.782 casos de personas contagiadas y 640 muertes ^2^^,^^4^. A diferencia del SARS-CoV, la transmisión de MERS-CoV esta geográficamente limitada ^4^, quizás porque no se transmite tan eficientemente de persona a persona como lo hace el SARS-CoV ^7^. De hecho, la mayoría de casos reportados de MERS-CoV provienen de brotes dentro de los países del Medio Oriente o de personas con viajes recientes a la región ^4^^).^ Se determinó que los dromedarios eran la fuente probable de infecciones humanas; sin embargo, posteriormente se descubrió que los murciélagos hospedaban virus estrechamente relacionados (similares al MERS), por lo que se supone que estos fueron la fuente evolutiva original ^2^.

Aunque no se ha confirmado la presencia de infección cerebral, las manifestaciones neurológicas asociadas con el MERS-CoV corresponden a estados mentales alterados que van desde confusión hasta coma, ataxia y déficits motores focales. Mediante resonancia magnética se han revelado lesiones bilaterales dentro de la sustancia blanca y las áreas subcorticales de los lóbulos frontal, temporal y parietal, así como en los ganglios basales, el cuerpo calloso, la protuberancia, el cerebelo y la médula cervical superior ^17^. En otros casos se ha reportado hemorragia craneal masiva espontánea con extensión intraventricular y hernia amigdalina sin que se haya evidenciado aneurisma, defectos estructurales, hipertensión, coagulopatía incontrolada o administración de antiplaquetarios, lo que sugiere la infección con MERS-CoV como causal ^18^. En algunos pacientes los signos incluyen encefalitis de Bickerstaff en superposición con el síndrome de Guillain-Barré y algunas otras neuropatías ^19^. En ratones transgénicos infectados con MERS-CoV se evidenciaron cambios histopatológicos caracterizados por manguitos perivasculares, degeneración celular y muerte neuronal con inclusiones citoplasmáticas basófilas prominentes, sobre todo en el tálamo y el tallo cerebral ^20^.

## La COVID-19 y el SARS-CoV-2

La pandemia de SARS-CoV-2 se originó al parecer en Wuhan, China, en diciembre de 2019 y, al igual que los virus ya mencionados, se cree que este provino de los murciélagos ^5^^,^^21^. El SARS-CoV-2 es sustancialmente diferente del SARS-CoV y el MERS-CoV en términos de las infecciones humanas. A diferencia de los otros virus, el SARS-CoV-2 es capaz de mantener una transmisión comunitaria sostenida y se estima que cada individuo infectado puede contagiar a otras dos o tres personas. Sin embargo, su tasa de letalidad es mucho más baja que la de SARS-CoV, del 9,6 % ^21^^,^^22^, en tanto que la del SARS-CoV-2 está entre 1 y 4,5 %. A mediados de agosto del 2020 se reportaban alrededor de 20 millones de casos confirmados, más de 730.000 muertes y la presencia del virus en 216 países, áreas o territorios ^23^.

El SARS-CoV-2 causa neumonía aguda muy letal, con signos clínicos similares a los reportados para el SARS-CoV y el MERS-CoV. Sin embargo, a diferencia del SARS-CoV, los pacientes infectados por el SARS-CoV-2 rara vez exhiben signos y síntomas prominentes en las vías respiratorias superiores, lo que indica que las células objetivo del SARS-CoV-2 pueden estar ubicadas en la vía aérea inferior ^9^, como sucede en la infección causada por el MERS-CoV ^24^.

El SARS-CoV-2 y el SARS-CoV tienen la capacidad de unirse a los receptores ACE2 ^25^^,^^26^. Esta enzima es un componente crucial del sistema renina-angiotensina (RAS). El eje regulador clásico RAS ACE-Ang II-AT1R y el eje contrarregulador ACE2 -Ang1-7-MasR tienen un papel esencial en el mantenimiento de la homeostasis en humanos. La ACE2 antagoniza la activación del sistema RAS clásico y protege contra el daño de los órganos en condiciones de hipertensión, diabetes y enfermedades cardiovasculares ^25^. En estudios recientes se ha demostrado mediante simulación computacional una mayor afinidad de la proteína del pico SARS-Cov-2 con el receptor ACE2 humano que la del pico Bat-CoV y el ACE2, lo que podría explicar la rápida propagación del SARS -CoV-2 en humanos ^26^.

Mediante análisis molecular se han postulado algunas diferencias asociadas con el receptor ACE2 que tendrían implicaciones en la patogenia del SARS -CoV-2 en diferentes poblaciones ^27^. No obstante, en otro estudio se concluyó que no hay diferencias en los niveles de expresión del receptor al comparar por sexo, edad y raza y, por ende, tampoco en el riesgo de adquirir la infección. En el mismo estudio se sugiere que sí parece existir una relación entre los niveles de expresión del ACE2 en los pulmones y un mayor riesgo de mortalidad por SARS-CoV y SARS-CoV-2 en los hombres que en las mujeres y en las personas de mayor edad ^28^.

Como ocurre con otros coronavirus respiratorios, el SARS-CoV-2 podría ingresar al sistema nervioso central por la ruta hemática atravesando la barrera hematoencefálica o, por vía nerviosa a través del sistema olfatorio ^29^^-^^31^. Esto último estaría respaldado por el hecho de que, con frecuencia, los pacientes de COVID-19 refieren pérdida del olfato ^29^ y hay evidencia de que las infecciones con coronavirus se diseminan principalmente desde el bulbo olfatorio hacia el tálamo y el tallo cerebral. La posible presencia del SARS-CoV-2 en los centros cardiorrespiratorios del tallo cerebral podría asociarse con las dificultades respiratorias ^30^^,^^31^.

El hallazgo del virus en una muestra de líquido cefalorraquídeo de un paciente con COVID-19 constituye una evidencia adicional de la presencia del SARS-CoV-2 en el sistema nervioso central ^32^. La ACE2 se expresa en las células gliales y en las neuronas, así como en las células endoteliales de los vasos sanguíneos del sistema nervioso, lo que las convierte en un objetivo potencial de la COVID-19 ^31^^,^^33^ independientemente de la ruta de ingreso del virus. Sin embargo, hasta el momento no hay estudios experimentales que evalúen las posibles vías de ingreso del virus al sistema nervioso central desde las estructuras periféricas o los mecanismos neurotrópicos subyacentes a la infección ^32^^,^^34^.

Recientemente se reportó que los pacientes con infección grave por SARS-CoV-2, especialmente de edad avanzada, presentaron diversas manifestaciones neurológicas asociadas con el sistema nervioso central, el periférico y el musculoesquelético. Estos pacientes tenían mayor probabilidad de desarrollar enfermedad cerebrovascular aguda, vasculitis, conciencia alterada y lesión muscular, así como mareos, hipogeusia, hiposmia, hipoxia, neuralgia y encefalopatía hipóxica ^30^^,^^32^^,^^35^^-^^42^. Por otra parte, algunos de los pacientes que presentan estado mental alterado parecen cumplir con condiciones clínicas de tipo psiquiátrico, como psicosis de nueva aparición, síndrome neurocognitivo asociado con demencia y trastorno afectivo ^43^. Aunque no se conocen estudios histopatológicos como los realizados con el SARS-CoV, las manifestaciones neurológicas sugieren que la COVID-19 podría tener efectos similares a los reportados en las infecciones con SARS-CoV o MERS-CoV.

Por ahora, las hipótesis de los mecanismos que producen los signos y síntomas asociados con la infección por SARS-CoV-2 se han enfocado en el daño neuronal y la reacción de las células gliales por la excesiva secreción de citocinas (tormenta de citocinas). Este fenómeno puede presentarse con edema cerebral e hipertensión intracraneal y con el daño de las células endoteliales y la consiguiente alteración de la permeabilidad de la barrera hematoencefálica. La infección también podría causar encefalitis autoinmunitaria por la presencia de autoanticuerpos que atacan las neuronas y células endoteliales de los vasos sanguíneos activando, así, el eje hipotalámico-hipofisario-glándulas suprarrenales, lo que desencadena estrés y otras alteraciones fisiológicas debido a la activación excesiva de los efectores de glucocorticoides y sus receptores ^39^^-^^42^.

Sin embargo, en pacientes con alteraciones neurológicas del sistema nervioso central se han reportado niveles más bajos de linfocitos y plaquetas y más altos de nitrógeno ureico en sangre en comparación con aquellos sin dichos signos neurológicos, pero no se han encontrado diferencias significativas en los exámenes de laboratorio de los pacientes con alteraciones del sistema nervioso central y de aquellos que no las tienen. Este fenómeno podría apuntar a la presencia de inmunosupresión en pacientes con COVID-19 que presentan signos neurológicos del sistema nervioso central ^29^.

Los estudios de muestras anatomopatológicas del virus aislado en el endotelio de la microcirculación cerebral, del líquido cefalorraquídeo y del tejido encefálico podrían contribuir a esclarecer su papel en el daño cerebral y su influencia sobre el centro cardiorrespiratorio en el tronco encefálico ^30^. Por ello es necesario hacer estudios experimentales *in vitro* y con modelos animales para descifrar los mecanismos patogénicos y fisiopatológicos de la infección con SARS-Cov-2.

Algunos autores han sugerido que el SARS-CoV-2 permanece latente en el sistema nervioso central durante mucho tiempo en los pacientes curados y que podría reactivarse posteriormente desencadenando enfermedades neurológicas ^44^. Después de la epidemia las secuelas neurológicas y neuromusculares asociadas con la COVID-19 podrían constituir una carga adicional para los sistemas de salud representada en costosos procesos de rehabilitación, y contribuir al incremento de la morbimortalidad debido a la aparición de diversos trastornos neurológicos.

Se ha sugerido, asimismo, que en algunos pacientes con manifestaciones neurológicas durante el período epidémico de la COVID-19 los médicos deben considerar la infección por SARS-CoV-2 como un diagnóstico diferencial para contribuir a prevenir la transmisión ^35^. Por ello se debe prestar atención a las manifestaciones neurológicas de los pacientes con COVID-19, especialmente las de aquellos con infecciones graves. También es importante hacer seguimiento a corto y mediano plazo de los pacientes dados de alta con signos neurológicos leves o moderados, pues podrían complicarse o tener efectos secundarios imprevisibles ^45^.
